# Psychological distress mediated the effects of self-stigma on quality of life in opioid-dependent individuals: A cross-sectional study

**DOI:** 10.1371/journal.pone.0211033

**Published:** 2019-02-06

**Authors:** Kun-Chia Chang, Chung-Ying Lin, Chih-Cheng Chang, Shuo-Yen Ting, Ching-Ming Cheng, Jung-Der Wang

**Affiliations:** 1 Jianan Psychiatric Center, Ministry of Health and Welfare, Tainan, Taiwan; 2 Department of Public Health College of Medicine, National Cheng Kung University, Tainan, Taiwan; 3 Department of Rehabilitation Sciences, Faculty of Health and Social Sciences, The Hong Kong Polytechnic University, Hong Kong; 4 Department of Psychiatry, Chi Mei Medical Center, Tainan, Taiwan; 5 Department of Senior Citizen Service Management, College of Recreation and Health Management, Chia Nan University of Pharmacy and Science, Tainan, Taiwan; 6 Tsaotun Psychiatric Center, Ministry of Health and Welfare, Nantou, Taiwan; 7 Departments of Internal Medicine and Occupational and Environmental Medicine, National Cheng Kung University Hospital, Tainan, Taiwan; University of South Florida, UNITED STATES

## Abstract

**Background:**

Both stigma and psychological distress affect quality of life (QOL). This study is an attempt to determine the effects of these two factors on QOL and to explore possible mediation effects between psychological distress and self-stigma in opioid-dependent individuals.

**Methods:**

This cross-sectional study comprised 268 consecutive, treatment-seeking opioid-dependent individuals who were interviewed using the brief version of the World Health Organization Quality of Life instrument (WHOQOL-BREF), the Self-Stigma Scale-Short (SSS-S), the Chinese Health Questionnaire-12 (CHQ-12), and the Opiate Treatment Index (OTI). A series of regression models were constructed to determine if the SSS-S and CHQ-12 predict the WHOQOL-BREF scores. Moreover, a comparison of the potential mediation effects of psychological distress (as assessed by the CHQ-12) was made between the SSS-S and the WHOQOL-BREF using the Baron and Kenny procedure (including three separate regressions), along with the Sobel test.

**Results:**

The CHQ-12 score was predictive of the scores for the four domains and almost all facets of the WHOQOL-BREF except the item, “Dependence on medical aids.” Nonetheless, the SSS-S score predicted three of the four facets of the social QOL after adjustment of the CHQ-12 score. Psychological distress completely mediated the relation between self-stigma and the physical, psychological, and environmental domains, and partially mediated the relationship between self-stigma and social QOL (two-tailed Sobel test: p = 0.02 for each domain).

**Conclusions:**

Psychological distress has a significant impact on the QOL of treated opioid users. It appears to be a core element in reducing the negative effects of self-stigma on aspects of QOL.

## Introduction

Psychological distress is quite common in opioid-dependent individuals because of the frequent psychological symptoms and stressful life events that occur during drug dependency [1]. In Taiwan, this issue may be more complicated because of the social context and drug policies. According to the Narcotic Exclusion Act enacted in 1955, the purer form of opioid-heroin is classified as schedule-1 drug and heroin users were viewed as criminals. On arrestment, they are prosecuted, sentenced and imprisoned, with no medical based programs provided. In 1998, the laws regarding illicit drug use (Drug Control Act) underwent major revision and since then the offenders have been dually identified as criminals and patients. Thereafter, short-term admission for opioid detoxification with 7–10 days of hospitalization, is available in several psychiatric centers. Opioid-dependent individuals usually do not seek for medical helps because they need to pay at least US$1,000 dollars for one course of this short-term treatment. It was not until 2006 that considerable changes have occurred in treatment and care structure in response to HIV epidemic; the Taiwan Center for Disease Control (CDC) began to permit methadone and publicly-funded buprenorphine for long-term treatment (at least one year) with mostly free service for HIV-infected heroin users and partial copayment. Under this circumstance, more than 60% of co-existing psychiatric disorders and about 23.4% of depressive disorders have been reported to have occurred in opioid-dependent individuals [2].

Quality of life (QOL), defined broadly as an individuals’ perception of his/her position in life in terms of cultural, social, and environmental contexts [3], provides clinicians with a holistic view of an individual’s health condition [4] and has been suggested as an important indicator by which to evaluate the health condition of opioid-dependent individuals [5]. Unfortunately, the aforementioned psychological distress appears to have a detrimental impact on both overall QOL [5–8] and different domains of QOL among drug users [9, 10]. Previous studies on the Taiwanese population have shown that psychological symptoms influence every facet of the brief version of the World Health Organization Quality of Life (WHOQOL-BREF) measurements in both healthy workers and community-dwelling elderly groups [11, 12]. However, how psychological distress affects the QOL domains and facets of opioid-dependent individuals remains unclear.

Unlike many chronic diseases, opioid dependence impacts QOL not only because of its physical health burden but also because of social inclusion and self-determination [13]. Opioid-dependent individuals, in many cultures, are targets of bias in arenas such as employment, socialization, and healthcare treatment because their disease is socially undesirable or even discriminated against [14–17]. Social context plays an essential role in the formulation of stigma. In Taiwan, in past decades, opioid (heroin) use disorder was viewed as a behavioral problem, and half of Taiwan’s prisoners were illicit drug users because of the drug regulations in Taiwan. In 1995, Taiwan initiated the National Health Insurance (NHI) program, which excludes items directly related to opioid use disorder [18]. It was not until 2006 that the Taiwan CDC began to offer free, long-term opioid agonist treatment (OAT) to HIV-infected heroin users as a high priority. Under the financial and political support, there were more than 100 sites and ten thousands of heroin users registered in OAT regularly. Moreover, clinicians have been required to upload prescription data of both methadone and public-funded buprenorphine to the national treatment system daily and no take-home dosages are permitted for methadone. Because opioid-dependent individuals are prone to develop stigma related to social contexts [19], it is necessary to tackle the challenge and assist recovery from opioid use disorder. On the other hand, stigma has been shown to impair QOL among illicit drug users [20–21], and it should thus be considered a predictor of the QOL score. Also, a high level of stigma has been associated with a high prevalence of psychological symptoms in opioid users [21–22]. Therefore, we hypothesized a mediation effect of psychological distress in the relationship between stigma and QOL among opioid-dependent individuals.

The present study, therefore, was aimed primarily at an analysis of the effects of psychological distress on QOL scores and further at an exploration of the possible mediating effect of psychological symptoms on the relationship between stigma and QOL scores in heroin users in Taiwan.

## Methods

### Research design

A cross-sectional design was employed in this study. It was approved by two institutional review boards (the Jianan Psychiatric Center, Ministry of Health and Welfare (IRB number: 14–022) and the Chi Mei Medical Center (IRB number: 10403–004), Tainan, Taiwan). To facilitate protection of privacy for vulnerable populations (including opioid users), we recruited participants at their most convenient locations, namely, two general hospitals and one psychiatry center. Among them, one general hospital and the Jianan Psychiatric Center were under the direct supervision of the Ministry of Health and Welfare and shared the same IRB. All eligible consecutive patients in the above three sites were invited to participate during the observation period. All participants were more than 20 years old, were diagnosed with opioid dependence by at least one psychiatrist using the DSM-IV (Fourth edition of Diagnostic and Statistical Manual of Mental Disorders) criteria, and all could read, speak, and understand spoken Mandarin Chinese or Taiwanese. All volunteered to participate and complete the assessments after the study purposes were explained to them. Opioid-dependent individuals with other OAT contraindications, such as severe liver disease or acute psychosis, were excluded.

### Participants and procedure

This cross-sectional study, conducted from April 2015 to August 2016, recruited consecutive treatment-seeking heroin users from three hospitals with the largest population of patients receiving long-term OAT (including methadone and buprenorphine treatment, but mainly methadone) in central and southern Taiwan. About 500 heroin users regularly received OAT at our recruitment sites (two general hospitals and one psychiatric center). The psychiatrist and case manager helped to identify potential candidates who met the recruitment criteria. Three research assistants were trained and mutually standardized for data collection. Participants were invited to join the study during their outpatient visits and/or their clinical services (i.e., daily observed dosage of methadone). After signing written informed consents, the eligible participants completed a background information sheet, the Self-Stigma Scale-Short (SSS-S), the Opiate Treatment Index (OTI), the Chinese Health Questionnaire-12 (CHQ-12), and the World Health Organization Quality of Life-Brief version (WHOQOL-BREF) under the supervision of the case managers. All the assessments were administered by asking all the participants to complete the questionnaires first independently followed by a standardized face-to-face procedure used to explain the meaning of the questions and assure the completeness and quality of the questionnaires.

### Instrumentation

#### Background information sheet

We collected the following demographics and clinical features from a self-reported background information sheet on the participants: age, which was classified into three groups (younger than 35 years, 35–50 years, and 50 years and older); education, which was classified as elementary school, junior high school and above; marital status, which was classified as married and not married; employment, which was classified as having a full-time job in the past 30 days or not; blood-borne infections including human immunodeficiency virus (HIV) and hepatitis C virus (HCV) were collected by self-report, and HIV infection was validated with a registration card allowing reimbursement for treatment by the Taiwan CDC; substance use history, including amphetamine use, was also collected by self-report.

#### Self-Stigma evaluation: Self-Stigma Scale-Short (SSS-S)

The 9-item SSS-S consists of three domains: cognition, affect, and behavior, with 3 items in each domain [23]. Each self-rated item uses a 4-point Likert scale ranging from 1 (strongly disagree) to 4 (strongly agree). By using a term for a minority group being tested, the SSS-S can be applied to different minority groups for evaluation of the perceived self-stigma in the past week. The psychometric properties of the SSS-S in the minority group classified as “people with mental illness” in Taiwan have been verified as satisfactory [24]. In the current study, we adopted the term “people who use heroin” in the SSS-S to specify our sampled participants, and we did not use “opioid-dependent individuals” to avoid any imposed stigma. A higher score in the SSS-S means a higher level of self-stigma, and a cutoff score above 2.5 suggests a high level of self-stigma. The Cronbach’s alpha was 0.895 in the current study.

#### Assessment of addiction severity under treatment: Opiate Treatment Index (OTI)

We used the Opiate Treatment Index (OTI) to measure 4 domains: heroin use, HIV risk-taking behavior, health status over the month preceding the assessment interview, and a social functioning scale covering the preceding 6 months [25]. A higher score in a domain indicates a more severe dysfunction for that particular domain. The content validity values of the Taiwan version of OTI were as follows: suitability, 4.66; clarity, 3.98, and usability, 3.80. The interrater reliability of these 4 domains were from 0.706 (social functioning) to 0.950 (HIV risk-taking behaviors), and their test-retest reliability were from 0.646 (HIV risk-taking behaviors) to 0.863(social functioning) [26]. In this study, the Cronbach’s alpha was 0.649 for social functioning, 0.752 for HIV risk-taking behaviors, and 0.890 for health status, respectively.

#### Assessment of psychological distress: Chinese Health Questionnaire-12 (CHQ-12)

The CHQ-12 is a validated questionnaire used to assess psychological distress derived from the General Health Questionnaire (GHQ), with the addition of specially designed, culturally relevant items [27]. The CHQ-12 emphasizes more somatic items than the GHQ since in Chinese culture, people tend to present depression and/or minor psychiatric symptoms with more somatic complaints. The CHQ-12 was validated for the Taiwanese context in both community and clinical settings [28], with the total score for all items ranging between 0 and 12, where a higher score represents a more severe degree of psychological distress in the past two weeks. Cheng et al. [29] previously demonstrated that the internal consistency measured by Cronbach’s alpha for the 12-item CHQ was about 0.84–0.83, while that measured in the present study was 0.90, indicating high internal consistency.

#### Measuring quality of life: The World Health Organization Quality of Life-Brief version (WHOQOL-BREF)

The WHOQOL-BREF Taiwan version measures QOL during the past two weeks using 26 items from the original WHOQOL-BREF and 2 culture-specific items for the Taiwanese population [30]. The WHOQOL-BREF Taiwan version contains two generic items (one measures overall QOL, and the other measures health) and four domains: physical (7 items), psychological (6 items), social relationships (4 items), and environment (9 items). Each self-rated item is scored from 1 to 5, and each domain score ranges from 4 to 20 through a linear transformation from the raw score, with a higher score indicating a better QOL. The WHOQOL-BREF Taiwan version has been proven to have satisfactory psychometric properties, with test–retest reliability coefficients within an interval of 2 to 4 weeks of 0.76 to 0.80 at all domain levels (p < 0.01) and an internal consistency (Cronbach’s α) coefficient of 0.91 for the entire questionnaire [30]. The Cronbach’s α in this study was 0.923. In addition, the WHOQOL-BREF was validated using a Rasch analysis for heroin users in our previous study [31].

### Statistical analysis

Before we constructed the multiple linear regression models intended to identify the sequential relationships among different levels of covariates with each WHOQOL-BREF domain and item, the collinearity among variables (e.g., age), and the OTI, SSS-S and CHQ-12 domains were assessed using Pearson’s correlation coefficients. The strongest correlation of 0.50 was between the two OTI domains (HIV risk behaviors and Heroin use Q score), and no significant multicollinearity was found between the variables included in the regression analysis. During the construction of the models, we included gender, age, education, marital status, employment status, blood-borne infections (HIV and/or HCV infection), concomitant substance use (amphetamine or benzodiazepine (BZD)), treatment status, OTI score, CHQ-12 score, and the SSS-S score as covariates. A series of regression models were applied to explore the relationship between the WHOQOL-BREF (including 26 items and four domains) and the determinants. Model 1 included all of the covariates listed above except the CHQ-12 score. Model 2 included all of the covariates except the SSS-S score, and Model 3 included all of the covariates.

**Fig 1:**
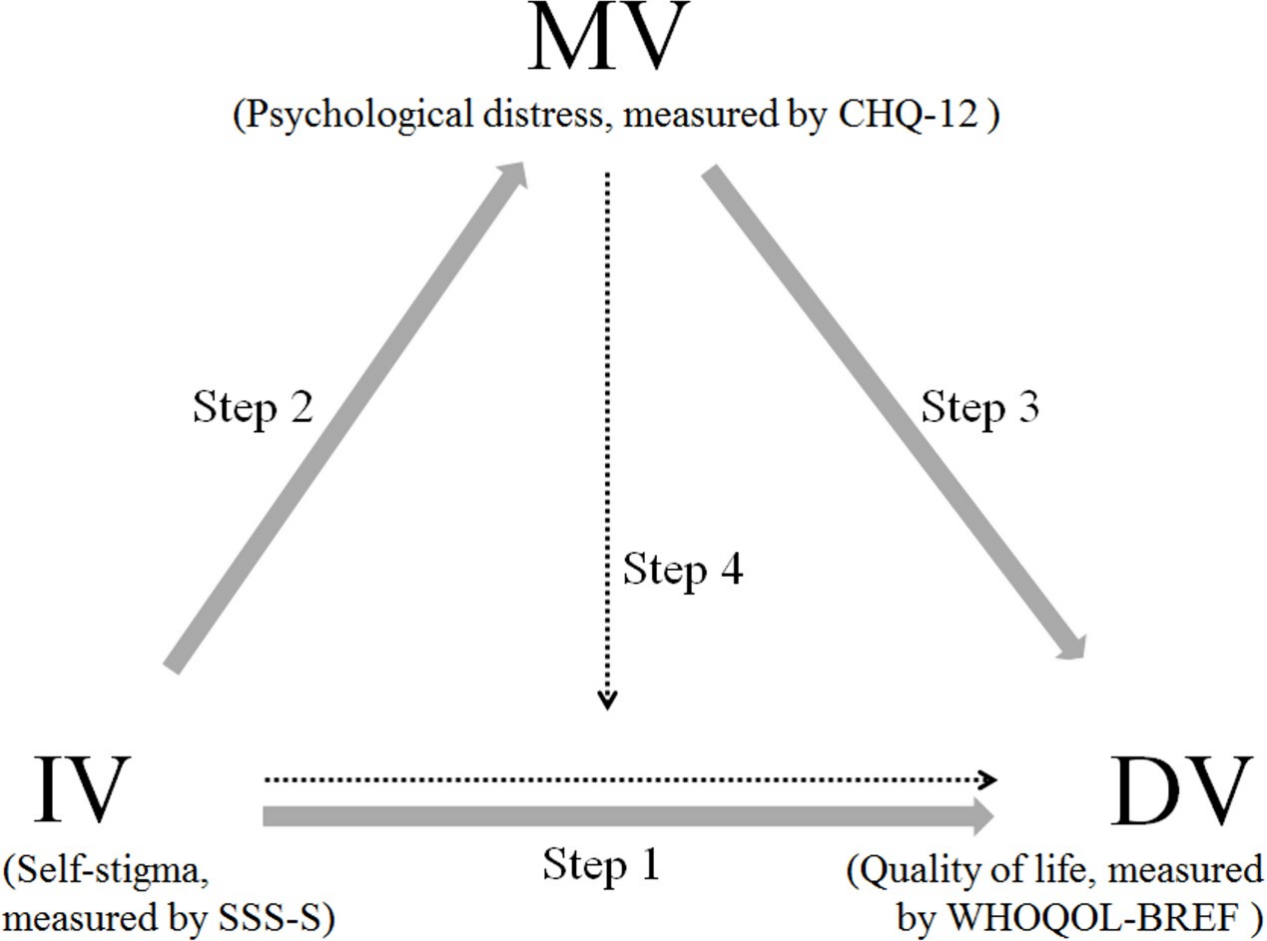
The procedure for testing of mediating effects. IV: independent variable; MV: mediating variable; DV: dependent variable. Step 1: to test if IV predicts DV without including MV as a covariate; Step 2: to test if IV predicts the MV; Step 3: to test if the MV predicts DV; Step 4: to test if IV predicts DV with MV as a covariate. In this study, psychological distress (measured by CHQ-12) was the MV, self-stigma (measured by SSS-S) was the IV, and Quality of life (measured by WHOQOL-BREF) was the DV. CHQ-12 = Chinese Health Questionnaire-12; SSS-S = the Self-Stigma Scale-Short; WHOQOL-BREF = the brief version of the World Health Organization Quality of Life instrument.

Next, to test the mediation effects of psychological distress, we applied a regression analysis, as recommended by Baron and Kenny [32]. According to their procedures, which are shown in Fig 1, in each regression model series, the variable order was as follows: In step 1, we examined the relationship between the independent variable (IV) and the dependent variable (DV), where the IV significantly predicted the DV. In step 2, the IV significantly predicted the mediating variable (MV). In step 3, the MV significantly predicted the DV. In step 4, after adjustment of the MV, the effect of the IV was either reduced (partial mediation) or was not significant (complete mediation). Multiple regressions controlling for gender, age, education, marital status, employment status, blood-borne infections, treatment status, heroin use, and four domains of the OTI were conducted. Any variable showing significant associations with each domain was identified as an IV and examined with the four criteria described above, where we assumed the CHQ-12 to be the MV. The significance of the estimated mediation effects was assessed with the Sobel test and the bootstrap method described by Preacher and Hayes (n = 5,000 bootstrapping samples) under the condition that any set of IVs and MVs within the respective domain score met the four criteria [33].

The statistical analysis was performed using the SAS statistical package (version 9.2 for Windows; SAS Institute, Inc., Cary, NC), and the significance level was set at α<0.05.

## Results

A total of 286 participants were recruited. However, only 268 (93.7%) completed the assessments for the analyses because 18 participants did not meet the inclusion criteria. Table 1 provides the participants’ characteristics and demographic data. Overall, the mean age was 44.9 ± 7.58 years; most of them were employed (72.4%), had contracted HCV (69.0%), and were male (88.8%). Only slightly more than twenty percent of them had remained married (22.4%), and 39 (14.6%) of the participants were HIV carriers. In addition, over eighty percent of the participants were currently enrolled in an OAT program (82.5%). Approximately two-thirds of the sample reported having used amphetamines, and 48 participants (27.6%) had used amphetamines in the past 4 weeks.

**Table 1:** Major determinants for WHOQOL-BREF domain scores under multiple linear regression models.

	N or mean±SD	†Phy Dom		†Psy Dom		†Soc Dom		†Env Dom	
		Est.(*SE*)	R^2^	Est.(*SE*)	R^2^	Est.(*SE*)	R^2^	Est.(*SE*)	R^2^
			0.61		0.46		0.42		0.42
**Gender**									
Female/male	30/238	-		-		-		-0.23(0.45)	
**Age**	44.9±7.58								
35-50/<35y	171/27	-0.34(0.41)		-		-		-	
≥50/<35y	70/27	0.3(0.47)		-		-		-	
**Education**									
6-9/≤6y	130/38	-0.3(0.36)		-0.67(0.43)		-		-0.11(0.41)	
≥9/≤6y	100/38	0.14(0.39)		-0.19(0.45)		-		0.74(0.43)	
**Marriage**									
Not married/married	208/60	-		-		-		-	
**Work**									
No/Yes	74/194	-1.08(0.28)***		-0.43(0.33)		-0.45(0.32)		-0.9(0.32)**	
**Human Immunodeficiency Virus**									
Yes/No	39/229	-0.13(0.34)		-0.7(0.4)		-0.5(0.38)		-0.34(0.38)	
**Hepatitis C virus**									
Yes/No	185/83	-0.22(0.27)		-0.13(0.32)		-		-0.22(0.31)	
**Amphetamine use history**									
Yes/No	174/94	0.02(0.26)		0.1(0.31)		-0.62(0.3)*		-	
**OAT**									
Yes/No	221/47	0.56(0.39)		0.26(0.47)		-0.46(0.44)		0.22(0.45)	
**OTI Heroin Q scores**	0.73±1.53	-0.19(0.09)*		-2.7*10^−3^(0.11)		-0.06(0.11)		-0.06(0.11)	
**Amphetamine use in the past 30 days**									
Yes/No	48/220	0.27(0.35)		-0.02(0.41)		0.31(0.4)		-0.14(0.4)	
**BZD use in the past 30 days**									
Yes/No	82/186	-0.21(0.27)		-0.08(0.32)		-0.06(0.31)		0.17(0.31)	
**OTI: HIV risk behavior scores**	4.51±5.02	0.03(0.03)		-0.02(0.03)		-0.03(0.03)		-0.02(0.03)	
**OTI: Social functioning scores**	16.27±7.00	-0.03(0.02)		-0.04(0.02)		-0.05(0.02)*		-0.01(0.02)	
**OTI: Health status scores**	7.75±4.70	-0.11(0.03)***		-0.11(0.04)**		-0.09(0.03)**		-0.06(0.04)	
**CHQ-12**	3.60±2.33	-0.63(0.06)***		-0.57(0.1)***		-0.48(0.09)***		-0.51(0.09)***	
**SSS-S**									
≥2.5/<2.5	219/49	-0.54(0.3)		-0.69(0.36)		-1.06(0.35)**		-0.67(0.35)	

*p<0.05

**p<0.01

***p<0.001

†Regression coefficients and standard errors (*SE*) of major determinants for WHOQOL-BREF domain scores under multiple linear regression models.

WHOQOL-BREF = the brief version of the World Health Organization Quality of Life instrument; OAT = Opioid Agonist Treatment; OTI = Opiate Treatment Index; CHQ-12 = Chinese Health Questionnaire-12; SSS-S = the Self-Stigma Scale-Short; Phy Dom = Physical domain; Psy Dom = Psychological domain; Soc Dom = Social relationship domain; Env Dom = Environmental domain

Over four-fifths (81.7%) of the sample had SSS-S scores above 2.5, for which the mean score for the participants was 2.9 ± 0.6. Table 1 also summarizes the regression coefficients in the multiple regression models for the scores for the different domains of the WHOQOL-BREF. The CHQ-12 score significantly predicted all four domains of the WHOQOL-BREF. Being employed was associated with higher scores in both the physical and environmental domains. There were significant associations between the OTI health status score and the scores for the three domains, with the exception of the environmental dimension. The SSS-S score was significantly associated with a lower domain score for social relationships. Also, the OTI social functioning scores had a significantly inverse relationship with the social domain of the WHOQOL-BREF.

The associations between the WHOQOL-BREF item and the SSS-S and CHQ-12 scores in three sequential models are summarized in Table 2. The regression coefficients for the CHQ-12 showed a reverse association with the scores for all items except for “Dependence on medical aids” when the CHQ-12 was included as one of the determinants. Nevertheless, the SSS-S was associated with four domains and 14 of the 26 items of the WHOQOL-BREF before the adjustment for the CHQ-12, whereas it was only associated with the scores of five items and one domain after the adjustment for the CHQ-12.

**Table 2:** The CHQ-12 and the SSS-S for individual facets and domains of the WHOQOL-BREF under multiple linear regression models†.

WHOQOL-BREF	Model1		Model2		Model3		
	SSS-S	R^2^	CHQ-12	R^2^	SSS-S	CHQ-12	R^2^
	Estimate(*SE*)		Estimate(*SE*)		Estimate(*SE*)	Estimate(*SE*)	
Overall QoL	-0.06(-0.12)	0.24	-0.15(0.03)***	0.35	0.04(0.11)	-0.15(0.03)***	0.35
General health	-0.32(0.14)*	0.28	-0.16(0.04)***	0.36	-0.19(0.12)	-0.16(0.04)***	0.36
**Physical**	-0.91(0.35)**	**0.48**	-0.66(0.07)***	**0.61**	-0.54(0.3)	-0.63(0.06)***	**0.61**
Pain and discomfort	0.43(0.19)*	0.16	-0.09(0.02)*	0.16	0.37(0.19)	-0.09(0.02)*	0.17
Dependence on medicinal substances and medical aids	0.26(-0.18)	0.17	0.03(-0.02)	0.16	0.25(0.19)	7.9*10-3(0.02)	0.17
Energy and fatigue	-0.35(0.14)*	0.31	-0.15(0.03)***	0.35	-0.23(0.13)	-0.12(0.03)***	0.36
Mobility	-0.13(-0.16)	0.21	-0.21(0.04)***	0.31	0.01(0.15)	-0.21(0.04)***	0.31
Sleep and rest	-0.27(-0.16)	0.26	-0.18(0.03)***	0.34	-0.12(0.14)	-0.18(0.03)***	0.34
Activities of daily living	-0.36(0.13)**	0.27	-0.19(0.04)***	0.36	-0.23(0.12)	-0.18(0.04)***	0.37
Work capacity	-0.09(-0.15)	0.27	-0.12(0.02)***	0.31	0.04(0.14)	-0.12(0.02)***	0.32
**Psychological**	-1.02(0.39)*	**0.36**	-0.61(0.09)***	**0.46**	-0.69(0.36)	-0.58(0.09)***	**0.46**
Positive feelings	-0.32(0.15)*	0.14	-0.11(0.03)***	0.17	-0.22(0.15)	-0.11(0.03)***	0.18
Spirituality/ religion/ personal beliefs	-0.13(-0.16)	0.16	-0.12(0.04)***	0.2	-0.03(0.15)	-0.12(0.04)**	0.2
Thinking/learning/memory/ concentration	-0.23(-0.15)	0.23	-0.9(0.03)**	0.25	-0.15(0.14)	-0.09(0.03)**	0.26
Bodily image and appearance	-0.58(0.15)***	0.19	-0.07(0.02)*	0.16	-0.53(0.15)***	-0.06(0.02)*	0.2
Self-esteem	-0.32(0.14)*	0.29	-0.22(0.04)***	0.39	-0.17(0.13)	-0.19(0.04)***	0.39
Negative feelings	-0.08(-0.18)	0.05	-0.09(0.03)*	0.08	-0.16(0.18)	-0.12(0.03)**	0.08
**Social relationships**	-1.35(0.37)***	**0.34**	-0.54(0.09)***	**0.41**	-1.06(0.35)**	-0.52(0.08)***	**0.43**
Personal relationships	-0.38(0.13)**	0.25	-0.16(0.04)***	0.31	-0.26(0.12)*	-0.16(0.03)***	0.33
Sexual activity	-0.22(-0.14)	0.22	-0.13(0.03)***	0.26	-0.13(0.14)	-0.13(0.03)***	0.27
Practical social support	-0.33(0.12)**	0.21	-0.12(0.03)***	0.25	-0.25(0.12)*	-0.12(0.03)***	0.26
Being respected	-0.56(0.13)***	0.28	-0.13(0.04)***	0.28	-0.47(0.12)***	-0.09(0.03)***	0.32
**Environment**	-0.97(0.38)*	**0.32**	-0.54(0.09)***	**0.41**	-0.67(0.35)	-0.52(0.08)***	**0.42**
Freedom, physical safety and security	-0.25(-0.16)	0.26	-0.19(0.04)***	0.33	-0.12(0.15)	-0.18(0.04)***	0.34
Physical environment (pollution/noise/ traffic/climate)	-0.38(0.15)*	0.22	-0.16(0.03)***	0.26	-0.26(0.14)	-0.16(0.03)***	0.27
Financial resources	-0.33(-0.17)	0.22	-0.13(0.04)**	0.25	-0.22(0.16)	-0.13(0.03)**	0.25
Opportunities for acquiring new information and skills	-0.16(-0.16)	0.11	-0.12(0.03)**	0.14	-0.09(0.16)	-0.12(0.03)**	0.14
Participation in and opportunities for recreation/leisure activities	-0.27(-0.18)	0.2	-0.09(0.02)*	0.22	-0.17(0.17)	-0.09(0.02)*	0.22
Home environment	-0.26(0.13)*	0.13	-0.12(0.03)***	0.19	-0.17(0.13)	-0.12(0.03)***	0.2
Health and social care: accessibility and quality	-0.26(0.11)*	0.2	-0.09(0.02)***	0.23	-0.18(0.11)	-0.09(0.02)**	0.24
Transport	-0.32(0.12)**	0.16	-0.12(0.03)***	0.21	-0.25(0.12)*	-0.12(0.03)***	0.22
Eating	-0.24(-0.16)	0.2	-0.21(0.04)***	0.29	-0.13(0.15)	-0.21(0.04)***	0.3

*p<0.05

**p<0.01

***p<0.001

†Model 1 adjusted for confounders of gender, age, education, work, HIV, HCV, OAT, OTI heroin Q scores, amphetamine use, BZD use, HIV risk behavior scores, social functioning scores, health status score, and self-stigma scale; Model 2 is adjusted for all confounders in Model 1 plus the psychological adjustment scores with the exception of the Self-Stigma Scale; Model 3 is adjusted for the same confounders as Model 1 plus the psychological adjustment scores.

HIV = Human Immunodeficiency Virus; HCV = Hepatitis C Virus; OAT = Opioid Agonist Treatment; OTI = Opiate Treatment Index; WHOQOL-BREF = the brief version of the World Health Organization Quality of Life instrument; CHQ-12 = Chinese Health Questionnaire-12; SSS-S = the Self-Stigma Scale-Short

We further tested the mediation effects of psychological distress (as assessed by the CHQ-12) in the relationship between the SSS-S and WHOQOL-BREF scores, for which the results are summarized in Table 3. The results of the Sobel test for mediation indicated that there were indirect associations between the SSS-S and all domains of the WHOQOL-BREF through the CHQ-12. Although the association between the SSS-S and the social relationship domain remained significant after adjusting for the CHQ-12, it appears that psychological distress might be the MV of the relationship between the SSS-S and all four domains of the WHOQOL-BREF. The formal two-tailed Sobel test (Table 3) demonstrated that the indirect effect was significant (p < 0.05) for all domains of the WHOQOL-BREF. The bootstrap results verified the Sobel test, with a bootstrapped 95% confidence interval around the indirect effect not containing zero, as shown in Table 3.

**Table 3:** The mediation effects of CHQ-12 between SSS-S and four domains of WHOQOL-BREF.

	Predictor	Outcome	β(*SE*)	95% CI	Standardized β	R^2^	Sobel test statistic, *p* value
Phy Dom							2.36, 0.018
	SSS-S	**(Direct effect)**	-0.54(0.3)	[-1.13, 0.06]	-0.07		
		**(Indirect effect)**	-0.37(0.17)*	[-0.72, -0.07]	-0.07		
Step 1	SSS-S	Phy Dom **(Total effect)**	-0.91(0.35)**	[-1.59, -0.22]	-0.12	0.48	
Step 2	SSS-S	CHQ-12	0.58(0.27)*	[0.04, 1.12]		0.49	
Step 3	CHQ-12	Phy Dom	-0.65(0.07)***	[-0.79, -0.51]		0.61	
Step 4	CHQ-12	Phy Dom	-0.63(0.07)***	[-0.77, -0.50]		0.61	
Psy Dom							2.35, 0.019
	SSS-S	**(Direct effect)**	-0.69(0.36)	[-1.41, 0.03]	-0.09		
		**(Indirect effect)**	-0.33(0.15)*	[-0.63, -0.05]	-0.05		
Step 1	SSS-S	Psy Dom **(Total effect)**	-1.02(0.39)*	[-1.79, -0.25]	-0.13	0.36	
Step 2	SSS-S	CHQ-12	0.58(0.27)*	[0.04, 1.12]		0.49	
Step 3	CHQ-12	Psy Dom	-0.59(0.08)***	[-0.75, -0.42]		0.46	
Step 4	CHQ-12	Psy Dom	-0.56(0.08)***	[-0.73, -0.40]		0.46	
Soc Dom							2.34, 0.019
	SSS-S	**(Direct effect)**	-1.06(0.35)**	[-1.76, -0.37]	-0.15		
		**(Indirect effect)**	-0.29(0.14)*	[-0.60, -0.04]	-0.05		
Step 1	SSS-S	Soc Dom **(Total effect)**	-1.35(0.37)***	[-2.09, -0.61]	-0.19	0.34	
Step 2	SSS-S	CHQ-12	0.58(0.27)*	[0.04, 1.12]		0.49	
Step 3	CHQ-12	Soc Dom	-0.53(0.08)***	[-0.69, -0.37]		0.41	
Step 4	CHQ-12	Soc Dom	-0.50(0.08)***	[-0.66, -0.34]		0.43	
Env Dom							2.34, 0.020
	SSS-S	**(Direct effect)**	-0.67(0.35)	[-1.36, 0.02]	-0.09		
		**(Indirect effect)**	-0.30(0.14)*	[-0.59, -0.05]	-0.05		
Step 1	SSS-S	Env Dom **(Total effect)**	-0.97(0.38)*	[-1.71, -0.23]	-0.14	0.32	
Step 2	SSS-S	CHQ-12	0.58(0.27)*	[0.04, 1.12]		0.49	
Step 3	CHQ-12	Env Dom	-0.54(0.08)***	[-0.70, -0.38]		0.41	
Step 4	CHQ-12	Env Dom	-0.52(0.08)***	[-0.68, -0.36]		0.42	

*p<0.05

**p<0.01

***p<0.001

Each regression was adjusted for confounders including gender, age, education, work, HIV, HCV, amphetamine, OAT, OTI heroin scores, amphetamine use, BZD use, HIV risk behavior scores, social functioning scores, and health status score.

HIV = Human Immunodeficiency Virus; HCV = Hepatitis C Virus; OAT = Opioid Agonist Treatment; OTI = Opiate Treatment Index; WHOQOL-BREF = the brief version of the World Health Organization Quality of Life instrument; CHQ-12 = Chinese Health Questionnaire-12; SSS-S = the Self-Stigma Scale-Short; Phy Dom = Physical domain; Psy Dom = Psychological domain; Soc Dom = Social relationship domain; Env Dom = Environmental domain

## Discussion

Although previous studies have examined psychological distress on the different domains of the WHOQOL-BREF among opioid-dependent individuals [9–10], this study is the first attempt to explore psychological distress (as assessed by the CHQ-12) and self-stigma (as assessed by the SSS-S) on every domain and facet of the WHOQOL-BREF. Hatzenbuehler [34] suggested that the effort required to cope with stigma in sexual minority groups diminishes individual psychological resources and therefore produces greater symptoms of psychological distress. The combined stress can result in adverse effects on both mental and physical health. Since treatment modalities, the access threshold and policy regulations vary across different countries, our study focus on the opioid users seeking for OAT, which is the mainstay of addiction treatment both in Taiwan and worldwide. Our findings suggest that in drug dependency groups, psychological distress may completely mediate the relation between self-stigma and the physical, psychological, environmental domains, and may partially mediate the relationship between self-stigma and social QOL, thus corroborating the hypothesis.

Our results indicated a strong relationship between QOL and psychological distress among opioid users, which was consistent with previous articles [35]. Although the WHOQOL-BREF and CHQ-12 may share overlapping constructs for the psychological and physical domains, the fact that the latter is also significantly predictive of every facet of the social and environment domains of the former cannot be explained by the overlapping construct alone. However, psychological distress as measured by CHQ-12 in this study was associated with the scores of every domain and all facets of WHOQOL-BREF except those for the “Dependence on medical aids” item. The results echo previous Asian studies suggesting a high prevalence of co-morbid mood disorders associated with low healthcare utilization among patients undergoing opioid agonist therapy [36,37]. It should be considered the heroin users are dually identified as criminals and patients in Taiwan and a substantial proportion of OAT subjects have been arrested first then transferred to long-term treatment. These contexts could themselves worsen the problems of stigma. Since eighty percent of our study subjects suffered from high levels of self-stigma, namely, had SSS-S scores over 2.5, and stigma was found between healthcare workers and drug users [38], many such heroin users will avoid seeking care from conventional healthcare facilities [39] even when they suffer from health problems reflected in the CHQ-12.

Another important finding of our study was to demonstrate the relationship between self-stigma and QOL. Also, we further examined how psychological symptoms modify the effect of self-stigma on QOL. Self-stigma significantly affected every domain of the WHOQOL-BREF before adjustment for the CHQ-12 scale, but only scores for the social domain remained statistically significant after adjustment for the CHQ-12 score (Table 2). Moreover, the initial predictive effects of self-stigma on 14 of the 26 facets of the WHOQOL-BREF were reduced to five after the adjustment for the CHQ-12 score (Table 2). Three of four facets among the social domain still remained significant, which indicates the independent, detrimental effect of self-stigma on social relationship QOL. These findings corroborate those of prior studies on stigma and QOL among HIV-infected subjects [40–42].

We further tested the mediation effect of psychological distress between stigma and QOL using Baron and Kenny’s procedure [32] and the Sobel test [33]. Table 3 summarizes the results and corroborates the possible complete mediation effects of psychological distress (as assessed by the CHQ-12) on the relationship between the scores for stigma and those for the physical, psychological, and environmental domains of the WHOQOL-BREF. It also indicates a partial mediation effect on the relationship of psychological distress with stigma and the social domain of the WHOQOL-BREF. The R-square values of the regression equations were generally above 0.4 (Table 3), indicating a good explanation when using our predictors for the variance of each domain of QOL. Therefore, we tentatively concluded that the mediation effect exists and that the detailed mechanism deserves further investigation. While self-stigma is highly prevalent among opioid users and generally affects their QOL, this issue may be dealt with through proper management of psychological distress.

In this study, we excluded patients with contraindications of agonist therapy such as severe liver cirrhosis and acute psychosis to avoid potential confounding. We also controlled for the demographic factors of abuse of other substances (amphetamine and benzodiazepines) and co-existing infectious diseases (HIV and HCV infection) in our model construction. Our final models showed that perceived physical problems (assessed by the OTI health status domain) also had negative associations with three domains of the WHOQOL-BREF. This is comparable with the findings of a previous Asian study [8]. Generally, physical problems are the most frequent co-occurring disorders among opioid-dependent individuals because of their chronic drug use, unhealthy life style, and risky injection behavior. Our results indicate the complexity of co-morbidity associated with patients with opioid dependence.

Given the great impact of psychological distress on every domain of QOL among treatment-seeking opioid users, we also found employment to be independently associated with the physical and environment domain scores. This implies the importance of the reintegration of people with mental illness into society, as was emphasized in a previous study [43]. Moreover, our findings indicating that current injection of heroin among opioid users was only associated with the physical domain scores of the WHOQOL-BREF could be a result of the long-term (mean duration: 31.78 ± 21.67 months) treatment effect of agonists. This also implies that QOL measurements can be considered an alternative outcome for long-term addiction treatment.

Several limitations of this study should be mentioned. First, the cross-sectional study design limits the power of causal inference. Additional longitudinal studies combined with repeated measurements are warranted to further examine the temporal relationships associated with the hypothesis of a mediation effect. Second, relatively few opioid-dependent subjects have access to opioid detoxification because the short-term hospitalization must be paid out-of-pocket and usually not affordable. Therefore, our study subjects were recruited from three largest long-term OAT sites in Taiwan as a convenience sample, and none of them were homeless at the time of the study. Although the characteristics of our sample were similar to those of Taiwanese nationally representative data [44], the treatment access threshold or policies that guarantee accessibility vary across different countries and one must be still cautious in generalizing our results. Third, the instrument (SSS-S) used for the self-stigma assessment was not designed specifically for opioid users. Instead, the SSS-S is an instrument widely applied in subjects with different conditions [45] based on its replaceable terms describing stigmatized populations. However, using the SSS-S offers the advantage of comparing populations with dependence on different substances. Last but not least, the participants’ understanding of the subjective construct under evaluation could have changed over time (response shift) or recollection could have been inaccurate (recall bias), which could have biased our results because of the interrelated nature and different time references of the instruments used in this study. However, in Taiwanese culture, participants tend to underreport their psychological distress (as assessed by CHQ-12 in this study). Because we found a high level of self-stigma in this study, the mediation effect of psychological distress between self-stigma and quality of life would only be violated by response shift bias. Since the major questionnaires (namely, SSS-S, CHQ-12 and WHOQOL-BREF) in our mediation model had relatively short time references (one or two weeks), and these measures were found to have good internal consistencies, with Cronbach alpha scores around 0.900 in this study, we assume the above biases would be minimal.

## Conclusions

This study corroborates the hypotheses positing that self-stigma and psychological distress are key predictors associated with QOL among treated heroin users. Furthermore, psychological distress very likely plays a mediating role in the relationship between self-stigma and QOL. Given that self-stigma is particularly prevalent among opioid-dependent individuals, effectively managing their psychological symptoms may still help improve or maintain a good QOL. However, high levels of self-stigma also indicate poor social QOL after adjustment for psychological distress. A comprehensive approach combining mental health assessment and treatment and social reintegration programs should be considered to alleviate psychological symptoms and reduce self-stigma, thereby promoting the overall QOL of opioid-dependent individuals.

## Supporting information

S1 Appendix Specific SAS procedure used for mediation analysis (SAS code_20180925.docx). (DOCX)

S2 Appendix Specific SAS procedure used for mediation analysis (SAS code.sas). (SAS)

## References

[1] Darke S, Ross J. Suicide among heroin users: rates, risk factors and methods. Addiction. 2002; 97(11):1383–1394. 10.1046/j.1360-0443.2002.00214.x 12410779

[2] Chen CC, Tsai SY, Su LW, Yang TW, Tsai CJ, Hwu HG. Psychiatric comorbidity among male heroin addicts: differences between hospital and incarcerated subjects in Taiwan. Addiction. 2002; 94(6):825–832. 10.1046/j.1360-0443.1999.9468256.x 10665072

[3] Skevington SM, Lotfy M, O'Connell KA. The World Health Organization's WHOQOL-BREF quality of life assessment: psychometric properties and results of the international field trial. A report from the WHOQOL group. Quality of Life Research. 2004; 13(2):299–310. 10.1023/B:QURE.0000018486.91360.00 15085902

[4] Su CT, Ng HS, Yang AL, Lin CY. Psychometric evaluation of the Short Form 36 Health Survey (SF-36) and the World Health Organization Quality of Life Scale Brief Version (WHOQOL-BREF) for patients with schizophrenia. Psychological Assessment. 2014; 26(3):980–989. 10.1037/a0036764 24796341

[5] De Maeyer J, Vanderplasschen W, Lammertyn J, van Nieuwenhuizen C, Sabbe B, Broekaert E. Current quality of life and its determinants among opiate-dependent individuals five years after starting methadone treatment. Quality of Life Research. 2011; 20(1):139–150. 10.1007/s11136-010-9732-3 20740316 PMC3023858

[6] Carpentier PJ, Krabbe PF, van Gogh MT, Knapen LJ, Buitelaar JK, de Jong CA. Psychiatric comorbidity reduces quality of life in chronic methadone maintained patients. American Journal on Addictions. 2010; 18(6):470–480. 10.3109/10550490903205652 19874168

[7] Colpaert K, De Maeyer J, Broekaert E, Vanderplasschen W. Impact of addiction severity and psychiatric comorbidity on the quality of life of alcohol-, drug-and dual-dependent persons in residential treatment. European Addiction Research. 2013; 19(4):173–183. 10.1159/000343098 23257413

[8] Iskandar S, van Crevel R, Hidayat T, Siregar IM, Achmad TH, van der Ven AJ, De Jong CA. Severity of psychiatric and physical problems is associated with lower quality of life in methadone patients in Indonesia. American Journal on Addictions. 2013; 22(5):425–431. 10.1111/j.1521-0391.2013.00334.x 23952886

[9] Chen YZ, Huang WL, Shan JC, Lin YH, Chang HC, Chang LR. Self-reported psychopathology and health-related quality of life in heroin users treated with methadone. Neuropsychiatric disease and treatment. 2012; 9:41–48. 10.2147/NDT.S37284 23293525 PMC3534320

[10] Teoh Bing Fei J, Yee A, Habil MH. Psychiatric comorbidity among patients on methadone maintenance therapy and its influence on quality of life. American Journal on Addictions. 2016; 25(1):49–55. 10.1111/ajad.12317 26692463

[11] Chang YC, Yao G, Hu SC, Wang JD. Depression affects the scores of all facets of the WHOQOL-BREF and may mediate the effects of physical disability among community-dwelling older adults. PLOS ONE. 2015; 10(5):e0128356 10.1371/journal.pone.0128356 26010571 PMC4444229

[12] Lu IC, Jean MCY, Lei SM, Cheng HH, Wang JD. BSRS-5 (5-item Brief Symptom Rating Scale) scores affect every aspect of quality of life measured by WHOQOL-BREF in healthy workers. Quality of Life Research. 2011; 20(9):1469–1475. 10.1007/s11136-011-9889-4 21431460 PMC3199547

[13] De Maeyer J, Vanderplasschen W, Broekaert E. Exploratory study on drug Users’ perspectives on quality of life: more than health-related quality of life?. Social Indicators Research. 2009; 90(1):107–126. 10.1007/s11205-008-9315-7

[14] Brown SA. Standardized measures for substance use stigma. Drug and Alcohol Dependence. 2011; 116(1–3):137–141. 10.1016/j.drugalcdep.2010.12.005 21257274

[15] Earnshaw V, Smith L, Copenhaver M. Drug addiction stigma in the context of methadone maintenance therapy: an investigation into understudied sources of stigma. International Journal of Mental Health and Addiction. 2013; 11(1):110–122. 10.1007/s11469-012-9402-5 23956702 PMC3743126

[16] Luo T, Wang J, Li Y, Wang X, Tan L, Deng Q, et al Stigmatization of people with drug dependence in China: a community-based study in Hunan province. Drug and Alcohol Dependence. 2013; 134(1):285–289. 10.1016/j.drugalcdep.2013.10.015 24239068

[17] Luoma JB, Twohig MP, Waltz T, Hayes SC, Roget N, Padilla M, Fisher G. An investigation of stigma in individuals receiving treatment for substance abuse. Addictive Behaviors. 2007; 32(7):1331–1346. 10.1016/j.addbeh.2006.09.008 17092656

[18] Fan CY, Tan HK, Chien IC, Chou SY. Prevalence of psychiatric disorders among heroin users who received methadone maintenance therapy in Taiwan. The American Journal on Addictions. 2013; 23: 249–256. 10.1111/j.1521-0391.2014.12090.x 24724882

[19] Simmonds L, Coomber R. Injecting drug users: a stigmatised and stigmatising population. International Journal on Drug Policy. 2009; 20(2):121–130. 10.1016/j.drugpo.2007.09.002 17981451

[20] Ahern J, Stuber J, Galea S. Stigma, discrimination and the health of illicit drug users. Drug and Alcohol Dependence. 2007; 88(2–3):188–196. 10.1016/j.drugalcdep.2006.10.014 17118578

[21] Van Nguyen H, Nguyen HLT, Mai HT, Le HQ, Tran BX, Hoang CD, et al Stigmatization among methadone maintenance treatment patients in mountainous areas in northern Vietnam. Harm Reduction Journal. 2017; 14(1):1 10.1186/s12954-016-0127-9 28056990 PMC5217586

[22] Tran BX, Vu PB, Nguyen LH, Latkin SK, Nguyen CT, Phan HT, et al Drug addiction stigma in relation to methadone maintenance treatment by different service delivery models in Vietnam. BMC public health. 2016;16(1):238 10.1186/s12889-016-2897-0 26956741 PMC4784456

[23] Mak WW, Cheung RY. Affiliate stigma among caregivers of people with intellectual disability or mental illness. Journal of Applied Research in Intellectual Disabilities. 2008; 21(6):532–545. 10.1111/j.1468-3148.2008.00426.x

[24] Wu TH, Chang CC, Chen CY, Wang JD, Lin CY. Further Psychometric Evaluation of the Self-Stigma Scale-Short: Measurement Invariance across Mental Illness and Gender. PLOS ONE. 2015; 10(2):e0117592 10.1371/journal.pone.0117592 25659115 PMC4320062

[25] Darke S, Hall W, Wodak A, Heather N, Ward J. Development and validation of a multidimensional instrument for assessing outcome of treatment among opiate users: the Opiate Treatment Index. Addiction. 1992; 87(5):733–742. 10.1111/j.1360-0443.1992.tb02719.x 1591524

[26] Chou SY, Chan HY. The reliability and validity of the modified chinese version of the opiate treatment index. Taiwanese Society of Psychiatry. 2015;29(1):51–58+iii.

[27] Cheng TA, Williams P. The design and development of a screening questionnaire (CHQ) for use in community studies of mental disorders in Taiwan. Psychological Medicine. 1986; 16(2):415–422. 10.1017/S0033291700009247 3726013

[28] Cheng TA. A community study of minor psychiatric morbidity in Taiwan. Psychological Medicine. 1988; 18(4):953–968. 10.1017/S0033291700009880 3270838

[29] Cheng TA, Wu JT, Chong MY, Williams P. Internal consistency and factor structure of the Chinese Health Questionnaire. Acta Psychiatrica Scandinavica. 1999;82:304–308. 10.1111/j.1600-0447.1990.tb01389.x 2260484

[30] Yao G, Chung CW, Yu CF, Wang JD. Development and verification of validity and reliability of the WHOQOL-BREF Taiwan version. Journal of the Formosan Medical Association. 2002; 101(5):342–351. 12101852

[31] Chang KC, Wang JD, Tang HP, Cheng CM, Lin CY. Psychometric evaluation, using Rasch analysis, of the WHOQOL-BREF in heroin-dependent people undergoing methadone maintenance treatment: further item validation. Health and Quality of Life Outcomes. 2014;12:148 10.1186/s12955-014-0148-6 25277717 PMC4190329

[32] Baron RM, Kenny DA. The moderator–mediator variable distinction in social psychological research: Conceptual, strategic, and statistical considerations. Journal of Personality and Social Psychology. 1986; 51(6):1173–1182. 10.1037/0022-3514.51.6.1173 3806354

[33] Preacher KJ, Hayes AF. SPSS and SAS procedures for estimating indirect effects in simple mediation models. Behavior Research Methods, Instruments, & Computers. 2004; 36(4):717–731. 10.3758/BF03206553 15641418

[34] Hatzenbuehler ML, Phelan JC, Link BG. Stigma as a fundamental cause of population health inequalities. American Journal of Public Health. 2013; 103(5):813–821. 10.2105/AJPH.2012.301069 23488505 PMC3682466

[35] De Maeyer J, Vanderplasschen W, Broekaert E. Quality of life among opiate-dependent individuals: A review of the literature. International Journal on Drug Policy. 2010; 21(5):364–380. 10.1016/j.drugpo.2010.01.010 20172706

[36] Chang KC, Wang JD, Saxon A, Matthews AG, Woody G, Hser YI. Causes of death and expected years of life lost among treated opioid-dependent individuals in the United States and Taiwan. The International Journal on Drug Policy. 2017; 43:1–6. 10.1016/j.drugpo.2016.12.003 28160734 PMC5420493

[37] Nguyen LH, Nguyen LHT, Boggiano VL, Hoang CD, Van Nguyen H, Le HT, et al Quality of life and healthcare service utilization among methadone maintenance patients in a mountainous area of Northern Vietnam. Health and Quality of Life Outcomes. 2017; 15(1):77 10.1186/s12955-017-0633-9 28427471 PMC5399322

[38] McLaughlin D, Long A. An extended literature review of health professionals' perceptions of illicit drugs and their clients who use them. Journal of Psychiatric and Mental Health Nursing. 1996; 3(5):283–288. 10.1111/j.1365-2850.1996.tb00127.x 9004621

[39] Solomon SS, Hawcroft CS, Narasimhan P, Subbaraman R, Srikrishnan AK, Cecelia AJ, et al Comorbidities among HIV-infected injection drug users in Chennai, India. Indian Journal of Medical Research. 2008; 127(5):447–452. 18653907 PMC5638642

[40] Galvan FH, Davis EM, Banks D, Bing EG. HIV stigma and social support among African Americans. AIDS Patient Care STDS. 2008; 22(5): 423–436. 10.1089/apc.2007.0169 18373417 PMC2831751

[41] Smith R, Rossetto K, Peterson BL. A meta-analysis of disclosure of one's HIV-positive status, stigma and social support. AIDS care. 2008; 20(10):1266–1275. 10.1080/09540120801926977 18608080

[42] Mak WW, Cheung RY, Law RW, Woo J, Li PC, Chung RW. Examining attribution model of self-stigma on social support and psychological well-being among people with HIV+/AIDS. Social Science & Medicine. 2007; 64(8):1549–1559. 10.1016/j.socscimed.2006.12.003 17223239

[43] Lv Y, Wolf A, Wang X. Experienced stigma and self-stigma in Chinese patients with schizophrenia. General Hospital Psychiatry. 2013; 35(1):83–88. 10.1016/j.genhosppsych.2012.07.007 22925273

[44] Lee CT, Chen VC, Tan HK, Chou SY, Wu KH, Chan CH, et al Suicide and other-cause mortality among heroin users in Taiwan: A prospective study. Addictive Behaviors. 2013; 38(10):2619–2623. 10.1016/j.addbeh.2013.03.003 23851391

[45] Mak WW, Cheung RY. Self-stigma among concealable minorities in Hong Kong: conceptualization and unified measurement. American Journal of Orthopsychiatry. 2010; 80(2):267–281. 10.1111/j.1939-0025.2010.01030.x 20553520

