# Supplementary material for: Psychological distress mediated the effects of self-stigma on quality of life in opioid-dependent individuals: A cross-sectional study
Source: PLoS One. 2019 Feb 6;14(2):e0211033. doi: 10.1371/journal.pone.0211033 (PMC6364895; doi:10.1371/journal.pone.0211033)
Supplement: S1 Appendix — (DOCX) [file pone.0211033.s001.docx]

/*model 1*/

**%macro** ***liner1_1***;

%do i= **1** %to **28**;

proc reg data=a.a7;

model Q&i.=sex_reg agegp_reg1 agegp_reg2 edu_reg1 edu_reg2 work_reg HIV HCV ampfrom_gp OSTfrom_gp Q_scores_of_heroin_use current_amphetamine_use_gp current_BZD_use_gp HIV_risk_taking_scores social_functioning_scores health_status_scores Stotal_reg;

run;

%end;

**%mend** ;

%***liner1_1***;

**%macro** ***liner1_2***;

%do i= **1** %to **4**;

proc reg data=a.a7;

model Dom&i.=sex_reg agegp_reg1 agegp_reg2 edu_reg1 edu_reg2 work_reg HIV HCV ampfrom_gp OSTfrom_gp Q_scores_of_heroin_use current_amphetamine_use_gp current_BZD_use_gp HIV_risk_taking_scores social_functioning_scores health_status_scores Stotal_reg;

run;

%end;

**%mend** ;

%***liner1_2***;

/*model 2*/

**%macro** ***liner2_1***;

%do i= **1** %to **28**;

proc reg data=a.a7;

model Q&i.=sex_reg agegp_reg1 agegp_reg2 edu_reg1 edu_reg2 work_reg HIV HCV ampfrom_gp OSTfrom_gp Q_scores_of_heroin_use current_amphetamine_use_gp current_BZD_use_gp HIV_risk_taking_scores social_functioning_scores health_status_scores psychological_adjustment_scores;

run;

%end;

**%mend** ;

%***liner2_1***;

**%macro** ***liner2_2***;

%do i= **1** %to **4**;

proc reg data=a.a7;

model Dom&i.=sex_reg agegp_reg1 agegp_reg2 edu_reg1 edu_reg2 work_reg HIV HCV ampfrom_gp OSTfrom_gp Q_scores_of_heroin_use current_amphetamine_use_gp current_BZD_use_gp HIV_risk_taking_scores social_functioning_scores health_status_scores psychological_adjustment_scores;

run;

%end;

**%mend** ;

%***liner2_2***;

/*model 3*/

**%macro** ***liner3_1***;

%do i= **1** %to **28**;

proc reg data=a.a7;

model Q&i.=sex_reg agegp_reg1 agegp_reg2 edu_reg1 edu_reg2 work_reg HIV HCV ampfrom_gp OSTfrom_gp Q_scores_of_heroin_use current_amphetamine_use_gp current_BZD_use_gp HIV_risk_taking_scores social_functioning_scores health_status_scores psychological_adjustment_scores Stotal_reg;

run;

%end;

**%mend** ;

%***liner3_1***;

**%macro** ***liner3_2***;

%do i= **1** %to **4**;

proc reg data=a.a7;

model Dom&i.=sex_reg agegp_reg1 agegp_reg2 edu_reg1 edu_reg2 work_reg HIV HCV ampfrom_gp OSTfrom_gp Q_scores_of_heroin_use current_amphetamine_use_gp current_BZD_use_gp HIV_risk_taking_scores social_functioning_scores health_status_scores psychological_adjustment_scores Stotal_reg;

run;

%end;

**%mend** ;

%***liner3_2***;
